# Seizure susceptibility to various convulsant stimuli in the BTBR mouse model of autism spectrum disorders

**DOI:** 10.3389/fphar.2023.1155729

**Published:** 2023-04-20

**Authors:** Martina Tallarico, Antonio Leo, Emilio Russo, Rita Citraro, Ernesto Palma, Giovambattista De Sarro

**Affiliations:** ^1^ Science of Health Department, School of Medicine and Surgery, Magna Graecia University of Catanzaro, Catanzaro, Italy; ^2^ System and Applied Pharmacology@University Magna Grecia, Science of Health Department, School of Medicine and Surgery, Magna Graecia University of Catanzaro, Catanzaro, Italy

**Keywords:** BTBR, GABA, glutamate, epilepsy, seizure, neuropsychiatric comorbidities

## Abstract

**Background:** Autism spectrum disorders (ASDs) are one of the most severe chronic childhood disorders in terms of prevalence, morbidity, and impact on society. Interestingly, several systematic reviews and meta-analyses documented a bidirectional link between epilepsy and ASD, supporting the hypothesis that both disorders may have common neurobiological pathways. According to this hypothesis, an imbalance of the excitatory/inhibitory (E/I) ratio in several brain regions may represent a causal mechanism underpinning the co-occurrence of these neurological diseases.

**Methods:** To investigate this bidirectional link, we first tested the seizure susceptibility to chemoconvulsants acting on GABAergic and glutamatergic systems in the BTBR mice, in which an imbalance between E/I has been previously demonstrated. Subsequently, we performed the PTZ kindling protocol to study the impact of seizures on autistic-like behavior and other neurological deficits in BTBR mice.

**Results:** We found that BTBR mice have an increased susceptibility to seizures induced by chemoconvulsants impairing GABA_A_ neurotransmission in comparison to C57BL/6J control mice, whereas no significant difference in seizure susceptibility was observed after administration of AMPA, NMDA, and Kainate. This data suggests that deficits in GABAergic neurotransmission can increase seizure susceptibility in this strain of mice. Interestingly, BTBR mice showed a longer latency in the development of kindling compared to control mice. Furthermore, PTZ-kindling did not influence autistic-like behavior in BTBR mice, whereas it was able to significantly increase anxiety and worsen cognitive performance in this strain of mice. Interestingly, C57BL/6J displayed reduced sociability after PTZ injections, supporting the hypothesis that a tight connection exists between ASD and epilepsy.

**Conclusion:** BTBR mice can be considered a good model to study epilepsy and ASD contemporarily. However, future studies should shed light on the mechanisms underpinning the co-occurrence of these neurological disorders in the BTBR model.

## 1 Introduction

Autism spectrum disorders (ASDs) are a set of neurodevelopmental conditions characterized by abnormal social communication and social interactions, repetitive behavior, and highly variable intelligence. ASD is often accompanied by neuropsychiatric comorbidities, such as intellectual disability (ID), epilepsy, anxiety, mood disorders, and cognitive deficits that further impair the quality of life (Lai et al., 2019; Damiani et al., 2020; Hirota and King, 2023). To date, in literature, several systematic reviews and meta-analyses have recognized, beyond doubt, that there is a more prevalent (12.1% and 17.2%) of epilepsy in patients with autism and *vice versa* (Strasser et al., 2018; Liu et al., 2022; Specchio et al., 2022).

The rate of ASD-epilepsy comorbidity is much higher, 21% in patients with intellectual disability and female gender, and 8% in those without intellectual disability vs. 0.8% in the general population (Anand and Jauhari, 2019; Besag and Vasey, 2021; Hirota and King, 2023). Numerous pieces of evidence indicate that an increased risk of epilepsy exists with increasing age in ASD patients, and the prevalence can double in adolescence (26%) compared to childhood (12%) (Sharma et al., 2022). Based on this overlapping prevalence, it has been suggested that ASD and epilepsy share at least some common pathogenic mechanisms. Hypothetical explanations to support the relationship between the two diseases comprise defects in synaptic growth, aberrant synaptic plasticity, and imbalance in neuronal excitation/inhibition (E/I) in various brain regions (Pan et al., 2021; Liu et al., 2022; Specchio et al., 2022; Zahra et al., 2022). Interestingly, several studies have reported that genetic, epigenetic, and/or environmental factors can converge, at the cellular level, to alter the E/I balance in different brain circuits, such as the neocortex, hippocampus, amygdala, and cerebellum, leading to ASD-epilepsy comorbidity (Bozzi et al., 2018; Cherubini et al., 2022; Specchio et al., 2022). Notably, it has been proposed that an increase in the ratio between E/I with consequent hyperexcitability in several brain circuits is a common mechanism in ASD that is responsible for the learning and memory, cognitive, sensory, motor deficits, and seizures occurring in these disorders (Nelson and Valakh, 2015; Uzunova et al., 2016). This imbalance between E/I seems to be primarily caused by deficits in GABA receptors, GABAergic synaptic neurotransmission, or maturation of GABAergic interneurons and their connections. Accordingly, dysfunction in the GABAergic system seems to provide a reasonable reason for the co-occurrence of epilepsy and ASD (Richard et al., 2017; Bozzi et al., 2018; Zhao et al., 2022). However, human genetics and experimental models studies have underlined that also glutamate dysfunctions, by increasing brain hyperexcitability, are implicated in ASD-epilepsy pathogenesis (Oberman, 2012; Bozzi et al., 2018; Chakraborty et al., 2022). Several experimental models mimicking behavioral phenotypes common to ASD display alterations in the E/I balance ratio (Bozzi et al., 2018; Zhao et al., 2022). To date, several aspects, including the link between the factors regulating the formation of E/I circuits during development, their impact on cellular and synaptic inhibition or excitation, and the risk for developing seizures and autism-like behavioral traits, remain to be established.

BTBR T + Itpr3tf/J (BTBR) mice are a well-recognized animal model of idiopathic autism, as having a compelling face validity for a range of behavioral impairments that are similar to those detected in patients with ASD, including reduced sociability, hyperactivity, increased repetitive/compulsive behaviors, and impaired ultrasonic vocalization (McFarlane et al., 2008; Meyza and Blanchard, 2017). Furthermore, the BTBR mice display other behavioral deficits observed in ASD, such as high anxiety and defective cognitive performance (Chao et al., 2018). BTBR mice also exhibit dysregulation of both the innate and adaptive systems and an inflammatory immune profile resembling ASD (Onore et al., 2013). Moreover, BTBR mice have prolonged intestinal transit, intestinal barrier dysfunction, and microbial dysbiosis symptoms similar to those reported in patients with ASD (Leo et al., 2021; Alamoudi et al., 2022).

Much of the BTBR phenotype is driven by several genetic and epigenetic alterations in the central nervous system (CNS). Furthermore, anatomical and functional alterations are also present in several brain regions, with the absence of a corpus callosum being the most notable abnormality, in BTBR mice (Mercier et al., 2012; McTighe et al., 2013; Meyza and Blanchard, 2017; Gandhi and Lee, 2021). Interestingly, an electrophysiological study has demonstrated that adult BTBR mice have a reduced amplitude of the spontaneous inhibitory postsynaptic current (IPSC), accompanied by a consequent increase in the frequency and amplitude of the excitatory postsynaptic current (EPSC), in the hippocampal slices compared to the control strain. The enhancement of the GABAA-mediated inhibition by low-dose of Clonazepam, reversing the deficit in IPSC, normalized autism-like behaviors in BTBR mice. By virtue of this, it has been proposed that an abnormal neuronal excitatory firing in specific brain regions caused by the absence of GABAergic inhibition underlies the autistic-like behaviors in BTBR mice (Gogolla et al., 2014; Han et al., 2014; Cellot et al., 2016; Gandhi and Lee, 2021). Furthermore, pharmacological evidence has also demonstrated that dysfunctions in the glutamatergic systems could be implicated in the etiology of ASD-like behavior in BTBR mice. In fact, glutamatergic drugs such as AMPAKINE, NMDAR modulators, and mGluR5 antagonists have been demonstrated to alleviate ASD symptoms (Silverman et al., 2012; Silverman et al., 2013; Nuzzo et al., 2020; Eissa et al., 2021). Of note, the disruption of the delicate balance between neuronal E/I is well documented to lead to seizure onset and epilepsy (Staley, 2015). Based on this background, the primary aim of this study was to evaluate seizure susceptibility in BTBR mice. Of note, BTBR mice do not exhibit an innate epileptic phenotype, and up to now, only a few studies have been focused on the seizure susceptibility of this strain (Ruskin et al., 2013; Lewis et al., 2018). Considering the high prevalence of epilepsy in ASD patients, we wished to define seizure susceptibility better and acquire further information to be used in future studies as a tool to study both drug effects and common mechanisms between ASD and epilepsy. Therefore, we initially determined the convulsive dose 50 s (CD_50_s) of several convulsant drugs with various known mechanisms of action (De Sarro et al., 2004a), including antagonists of GABA–benzodiazepine inhibitory–Cl^−^ ionophore receptor complex [i.e., bicuculline, picrotoxin, pentylenetetrazole (PTZ) and methyl-6,7-dimethoxy-4-ethyl-beta-carboline-3-carboxylate (DMCM)] and agonists of ionotropic glutamate receptors [i.e., N-methyl-d-aspartate (NMDA), α-amino-3-hydroxy-5-methyl-4-isoxazolepropionate (AMPA) and kainic acid (KA)]. The secondary aim was to perform the PTZ kindling protocol, a chronic model of epilepsy, to study the impact of seizures on autistic-like behavior and other neuropsychiatric deficits in BTBR mice.

## 2 Materials and methods

### 2.1 Animals

Male BTBR and C57BL/6J (B6) mice aged 4-5 weeks were purchased from the Jackson Laboratory (JAX, Bar Harbor, ME, United States) and were housed up to four per cage in a climate-controlled room with a reversed light cycle (lights on at 19:00 h, off at 07:00 h). Food and water were provided *ad libitum*. All experimental protocols and animal handling procedures were conducted in conformity with international and national law and policies (EU Directive 2010/63/EU for animal experiments, ARRIVE guidelines, and the Basel declaration, including the “3R” concept). The experimental protocols and the procedures reported herein were approved (Authorization n° 382/2019-PR) by the Animal Care Committee of the University of Catanzaro, Italy. All efforts were made to minimize animal suffering and to use only the number of animals necessary to produce reliable scientific data.

### 2.2 Experimental summary

We planned two different sets of experiments; in ***experiment #1,*** we evaluated seizure susceptibility and, therefore, brain excitability in autistic BTBR mice at 8 weeks of age compared to age-matched control B6 mice after acute injection of chemoconvulsants with different mechanisms of action. Male BTBR and B6 mice of 8 weeks of age were randomly divided into several groups and were tested with the following chemoconvulsants: GABA antagonists (i.e., bicuculline, picrotoxin, DMCM, PTZ); glutamate agonists (i.e., AMPA, NMDA, and Kainate). Convulsants were chosen according to the study of the potential alteration in these two neurotransmitter systems. The times and the route of administration of these compounds were selected according to other previous studies (De Sarro et al., 2004a; De Sarro et al., 2004b; Russo et al., 2004).

In ***experiment #2,*** we studied BTBR mice’s susceptibility to PTZ-kindling and its impact on animal behavior in a set of various tests evaluating different aspects spanning from sociability to anxiety and cognitive impairment (see Section Behavioral Tests). Regarding behavioral tests, for every single mice group, testing started 24 h after the last PTZ injection necessary to achieve the kindling criterion within the group (Nesci et al., 2020). Likewise, testing for non-kindled mice started contemporarily with their respective kindled control groups. In order to reduce the number of mice used and avoid the effect played by different tests in the same animal, kindled and non-kindled BTBR and B6 mice were randomly divided into two subgroups (*n* = 24). In detail, the first subgroup of kindled and non-kindled BTBR and B6 mice (*n* = 6 for each group) were subjected to the marble burying test (MB) and three-chamber test (TCT), whereas the second subgroup was subjected to the elevated plus maze (EPM) and passive avoidance test (PA).

### 2.3 Drugs

DMCM, NMDA, picrotoxin, AMPA, and Kainate were purchased by Tocris (Buckhurst Hill, United Kingdom), bicuculline, and PTZ from Sigma (St. Louis, MO, United States). For systemic injections, all compounds (picrotoxin, PTZ, DMCM, bicuculline) were given intraperitoneally (i.p.) at a volume of 0.1 mL/10 g of mouse body weight. For intracerebroventricular (i.c.v.) injections (NMDA, AMPA, or Kainate), mice were anesthetized with isoflurane, and injections were made in the left or right lateral ventricle (coordinates 1 mm posterior and 1 mm lateral to the bregma; depth 2.4 mm) using a 5-μL Hamilton microsyringe; the injections of the drug by this procedure led to a uniform distribution throughout the ventricular system within 10 min (De Sarro et al., 2004a). All solutions were freshly prepared before the experiments.

### 2.4 Experiment #1: Acute seizures models

#### 2.4.1 Picrotoxin-, bicuculline- and PTZ-induced seizures

Different convulsive doses of picrotoxin (0.5–4 mg/kg; *n* = 8 mice for each dose), bicuculline (0.5–5 mg/kg; *n* = 8 mice for each dose), or PTZ (30–80 mg/kg; *n* = 8 mice for each dose) were tested in BTBR and B6 mice. Immediately after the drug injection, mice were transferred to a transparent plexiglass cage and monitored for 30 min to observe the occurrence of acute seizures. A threshold convulsion has been considered an episode of clonic spasms lasting for at least 5 s. The absence of this threshold convulsion over 30 min indicated that the animal was protected from the chemo convulsants-induced acute seizures (De Sarro et al., 2004a; De Sarro et al., 2004b).

#### 2.4.2 DMCM-induced seizures

Clonic seizures were induced by administration of DMCM that was dissolved in a minimal amount (<5% of final volume) of glacial acetic acid, brought to volume with saline, and administered to BTBR and B6 mice at different doses (0.5–8 mg/kg: i.p. *n* = 8 mice for each dose). The animals were monitored for 30 min after the injection for the incidence of clonic seizures, as previously described (De Sarro et al., 2004a; De Sarro et al., 2004b).

#### 2.4.3 Glutamate receptor agonists-induced seizures

Seizures were induced by i.c.v. Injections of NMDA (0.5 and 10 nmol/mouse; *n* = 8 mice for each dose) and AMPA (0.5 and 10 nmol/mouse; *n* = 8 mice for each dose) in BTBR and B6 mice using a 5-μL Hamilton microsyringe. Subsequently, mice were observed for 30 min, as previously described (De Sarro et al., 2004a; De Sarro et al., 2004b). Kainate was dissolved in neutral-buffered saline and administered by i.p. injection at doses comprised between 10 and 25 mg/kg (*n* = 8 mice for each dose) in both mice strains as reported (De Sarro et al., 2004a; De Sarro et al., 2004b).

### 2.5 Experiment #2

#### 2.5.1 Development of PTZ-kindling

PTZ-kindling was induced by the i.p administration of sub-convulsive doses of PTZ (30 mg/kg in a volume of 10 mL/kg) every other day in BTBR and B6 mice, starting from 8 weeks of age up to kindling development as previously described (De Sarro et al., 2004a; Nesci et al., 2020). After PTZ injection, BTBR and B6 mice were placed individually into Plexiglas cages and monitored for 30 min to detect seizure scores using the following scale: 0 = no change in behavior; 0.5 = abnormal behavior (sniffing, extensive washing, orientation); l = isolated myoclonic jerks; 2 = atypical (unilateral or incomplete) clonic seizures; 3 = fully developed bilateral forelimb clonus; 3.5 = forelimb clonus with a tonic component and twist of the body; 4 = tonic-clonic seizure with suppressed tonic phase, only clonus of all limbs; and 5 = fully developed tonic-clonic seizures. Another group of BTBR and B6 received saline with the same number of injections, and they were used as control for behavioral tests. Animals were considered to be fully kindled if they had at least three consecutive stages 5 seizures (Russo et al., 2013; Nesci et al., 2020).

#### 2.5.2 Behavioral tests

All experimental groups were subjected to a single test per day. Moreover, when two tests were performed on the same mouse, at least 1 day (range 1–3 days) was allowed, as previously reported by (Leo et al., 2021). All behavioral tests, except for MB, were performed under controlled temperature, humidity, and light intensity (dim illumination) with the support of video-tracking software (EthoVision XT15; Noldus Information Technology, Wageningen, the Netherlands) (Citraro et al., 2019; Leo et al., 2021). Experiments were always carried out during the dark phase of the cycle (between 10:00 a.m. and 2:00 p.m.) to avoid possible circadian alterations.

#### 2.5.3 Marble burying test (MB)

MB test was performed for testing repetitive and compulsive behaviors, as previously described (Silverman et al., 2010; Leo et al., 2021). A Plexiglas cage (46 cm × 24 cm × 21 cm) filled with 5 cm of clean bedding was used. On the first day of testing, each mouse was individually habituated in the plastic cage for 30 min without marbles. Twenty-4 hours later, an identical plastic container was prepared with 5 cm of clean bedding and 20 glass marbles (1.5 cm diameter) that were evenly distributed on the top of the bedding, equidistant from each other in a symmetrical 4 × 5 cm grid. Mice were then singly placed in the plastic container and allowed to explore for 30 min the space and the marbles freely. At the end of the session, the mouse was gently removed, and the total number of marbles buried was recorded. To be considered buried, marble needed to be 75% covered by bedding (Leo et al., 2021).

#### 2.5.4 Three chamber test (TCT)

Social approach behaviors were tested in a three-chambered apparatus using methods similar to those previously described (Silverman et al., 2010; Chang et al., 2017). The device is a rectangular three chambers box (left, center, and right), separated by transparent Plexiglas dividing walls with a rectangular opening to allow the free movement of mice. Briefly, this test consists of 3 phases; the first one is a 10-min habituation to the empty arena. Thereafter, an unfamiliar adult animal was placed on the either (right or left) side of the compartment, inside a small wire cage, whereas an inanimate object was placed inside an identical wire cage in the opposite side chamber. The test mouse was again placed in the empty center chamber. The dividers are then raised, allowing the test subject to move freely throughout all three compartments of the apparatus over a 10-min test session. Normal social behavior was defined as the time spent by the subject mouse in the chamber containing the novel mouse vs. the time spent in the chamber containing the inanimate novel object. Equal or less time spent with the novel animal would represent the absence of sociability in this task. Based on this, the time spent in each chamber and the time spent sniffing the novel object, and the time spent sniffing the novel mouse during the 10 min test session was scored (Leo et al., 2021). After each session, the apparatus was systematically cleaned (70% ethanol) to remove olfactory cues.

#### 2.5.5 Elevated plus maze test (EPM)

The EPM consists of two opposing open arms (45 × 10 cm) and two opposing closed arms (45 × 10 cm) of the same size, with 10 cm high walls connected by a central platform (10 × 10 cm) and elevated 80 cm above the floor. Each mouse was placed in the center of the maze facing the closed arm; during a 10 min observation period, the amount of time spent in the open arm, closed arm, and in the central platform was recorded as previously described (Leo et al., 2021). The shorter the time spent in the open arm and central platform, the higher the anxiety is and *vice versa*. The latency to first entry in open arms, mean velocity, and total distance moved were also measured and examined for every experimental group. After each session, the apparatus was systematically cleaned (70% ethanol) to remove olfactory cues.

#### 2.5.6 Passive avoidance (PA)

PA is a fear-motivated test performed to investigate learning and memory in rodents. In this test, mice learn to restrain their innate tendency, namely, preferring a dark compartment rather than an illuminated one (Zovkic and Sweatt, 2013). The PA test was conducted over two consecutive days, as previously reported (Leo et al., 2019; Leo et al., 2021). The latency to enter (s) in the dark compartment was recorded and analyzed. Briefly, on day one (habituation), mice were placed individually in the light chamber and allowed to explore the cage freely. Subsequently, 15 min later, the conditioning or learning task was initiated. Briefly, mice were individually placed in the light compartment with the sliding door closed. Following 30 s of delay, this door, separating the two compartments, was automatically opened. When the mouse entered into the dark chamber, an electrical foot shock (0.5 mA for 3 s) was administered by the floor grid. The latency to enter (s) in the dark compartment was recorded and analyzed. The retention trial was carried out 24 h (day two) after the acquisition trial by reintroducing the mouse into the light chamber of the cage. The mouse’s memory was evaluated by recording the latency (s) to enter into the dark compartment; however, no foot shock was administered. The cutoff time for each session was 300 s. Retention memory is directly linked to the latency to enter into the dark compartment: the better the memory, the greater the latency (Nesci et al., 2020; Leo et al., 2021). After each trial, the maze was systematically cleaned to eradicate olfactory cues (70% ethanol).

### 2.6 Statistical analysis

The differences between BTBR and B6 mice in the seizure incidence phase were analyzed using Fisher’s exact probability test. The percentages of mice exhibiting clonic or tonic seizures following various chemoconvulsants were plotted against the corresponding doses by a computer construction of the dose-effect curves for calculation of CD50 (±95% confidence limits). The CD50 values for each compound were calculated using the method of Litchfield and Wilcoxon (1949) by using a commercial computer program (PHARM/PCS, version 4.2). Outcomes, in the PTZ-kindling, were analyzed by two-way ANOVA followed by Sidak’s *post hoc* test (Nesci et al., 2020; Javaid et al., 2023). Likewise, data obtained from behavioral tests were analyzed by two-way analysis of variance (ANOVA) followed by Sidak’s *post hoc* test with strain (two levels) and kindling (two levels) as factors*.* Data were expressed as means ± S.E.M. p < 0.05 was considered significant. All statistical procedures were carried out using GraphPad Prism 9.0 (GraphPad Software, Inc., La Jolla, CA, United States).

## 3 Results

### 3.1 Acute effects of chemoconvulsants on seizure susceptibility

BTBR mice have shown a higher susceptibility to chemoconvulsants impairing the GABA_A_ neurotransmission, compared to B6 mice, except for the convulsion induced by the administration of picrotoxin (Table 1). In detail, BTBR mice showed a significant reduction of the PTZ CD_50_ (34.82 mg/kg) compared to B6 mice (48.32 mg/kg). Likewise, BTBR mice have also a decreased bicuculline and DMCM CD_50_ (1.82 and 1.95 mg/kg, respectively) compared to B6 mice (3.38, and 3.5 mg/kg, respectively), indicating a decreased seizure threshold (Table 1). At odds, no difference in seizure susceptibility to glutamatergic agonists was found between BTBR and B6 mice groups (Table 2).

**TABLE 1:** Seizure susceptibility of C57BL/6J and BTBR mice to convulsant drugs acting on the *γ*-aminobutyric acid–benzodiazepine receptor A. All data reported in the present table are expressed as mg/kg i.p.

Mouse strain convulsant	Dose	CD50 values (± 95% confidence limits)	
		C57BL/6J	BTBR
Picrotoxin	1, 1.5, 2, 3, 3.5, 4	2.5 (1.98–3.17)	2.2 (1.9–2.5)
Bicuculline	1, 2, 2.5, 3, 3.5, 4, 5	3.38 (3.08–3.93)	1.82 (1.45–2.28)*
Pentylenetetrazole	20, 30, 40, 50, 60, 70, 80	48.32 (42.73–54.63)	34.82 (29.43–41.20)*
DMCM	2, 2.5, 3, 4, 4.5, 5	3.5 (2.96–4.13)	1.95 (1,47–2,59)*

*p < 0.05 vs. C57BL/6J

**TABLE 2:** Seizure susceptibility of C57BL/6J and BTBR mice to convulsant drugs acting on the inotropic glutamatergic receptors. All data reported in the present table are expressed as nmol/mouse except for kainate which is expressed as mg/kg.

Mouse strain convulsant	Dose	CD50 values (± 95% confidence limits)	
		C57BL/6J	BTBR
NMDA	0.5–10	2.14 (1.62–2.83)	2.38 (1.70–3.33)
AMPA	0.5–10	2.42 (1.72–3.40)	2.16 (1.44–3.24)
Kainic acid	10–25	0.045 (0.037–0.055)	0.041 (0.033–0.051)

### 3.2 Evaluation of PTZ-induced seizure susceptibility

**FIGURE 1:**
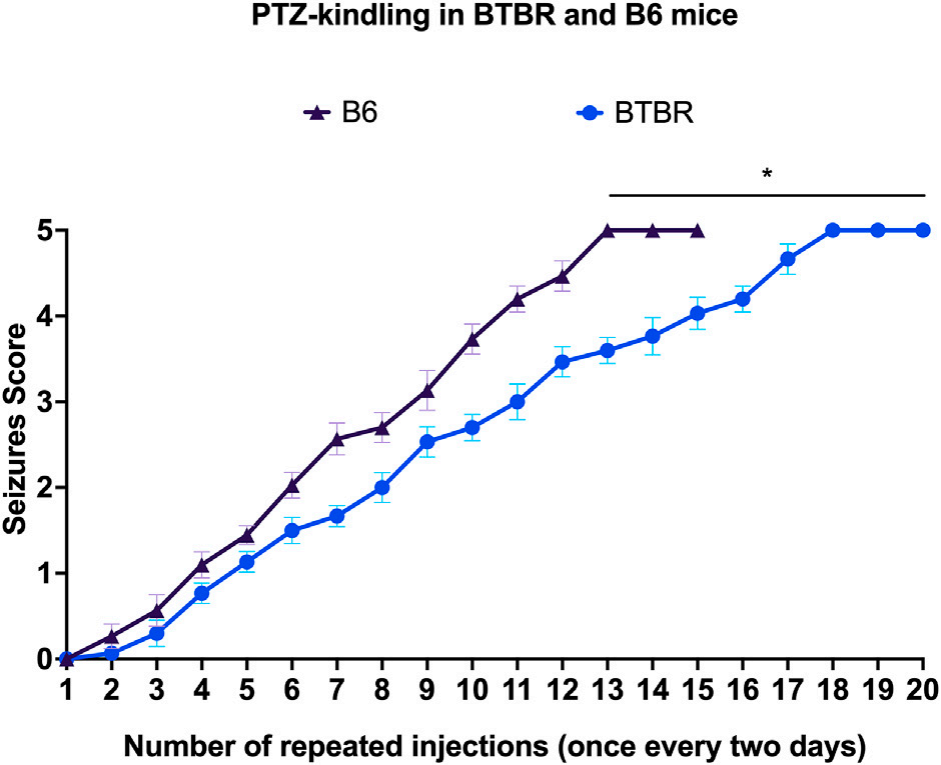
PTZ kindling development. Progression of kindling in B6 and BTBR mice. ^*^Indicates *p* < 0.01 vs. B6 mice. B6 = C57BL/6J; PTZ = Pentylentetrazole.

Kindling seizure development was progressive and directly proportional with the repeated administration of PTZ, and none of the mice convulsed on the first injection. Behavioral changes of PTZ kindling mice are shown in Figure 1. Surprisingly, B6 mice kindled before BTBR mice (*p* < 0.01). We observed that the 3rd stage 5 seizure was reached after 15 PTZ injections in B6 mice and after 20 PTZ injections in BTBR mice, indicating that this latter strain of mice is more resistant to PTZ kindling than B6 mice (Figure 1).

### 3.3 Effects of PTZ kindling on repetitive behavior and social interaction

**FIGURE 2:**
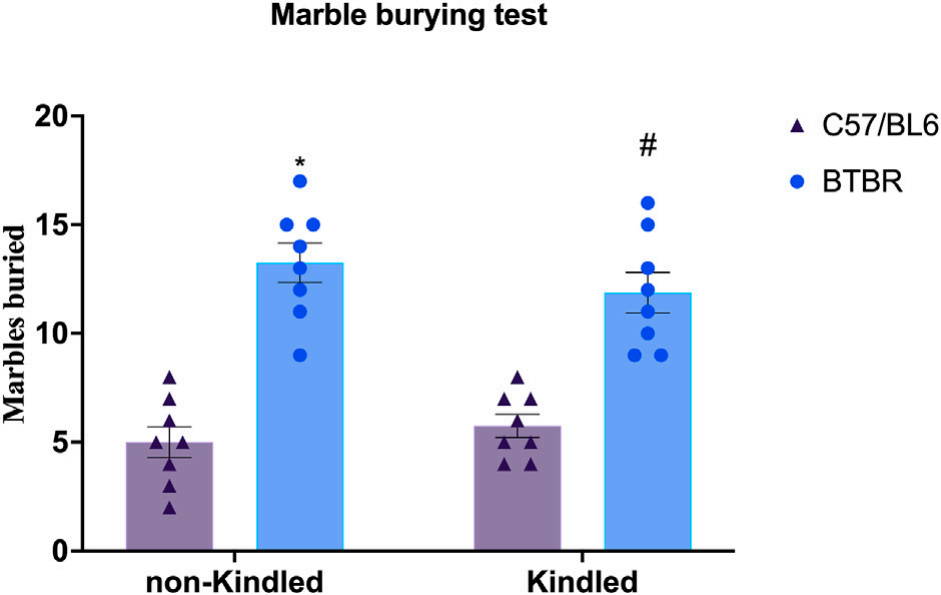
Marble burying assay performed at the end of the PTZ-kindling procedure. Bars indicate the number of marbles buried. Data are expressed as means ± S.E.M. ^*^Indicates *p* < 0.0001 vs. non-kindled B6 mice, ^#^Indicates *p* < 0.0001 vs. kindled B6 mice. B6 = C57BL/6J; PTZ = Pentylentetrazole.

BTBR mice exhibit repetitive/compulsive behaviors and impaired sociability, indicating an autistic-like phenotype (Silverman et al., 2010; Chang et al., 2017). Not surprisingly, in the MB test, we observed that BTBR mice buried significantly (*p* < 0.0001) more marbles than their respective B6 control mice, confirming a stereotyped behavior. PTZ-kindling did not significantly (*p* > 0.05) modify this parameter neither in BTBR nor in B6 mice (Figure 2).

**FIGURE 3:**
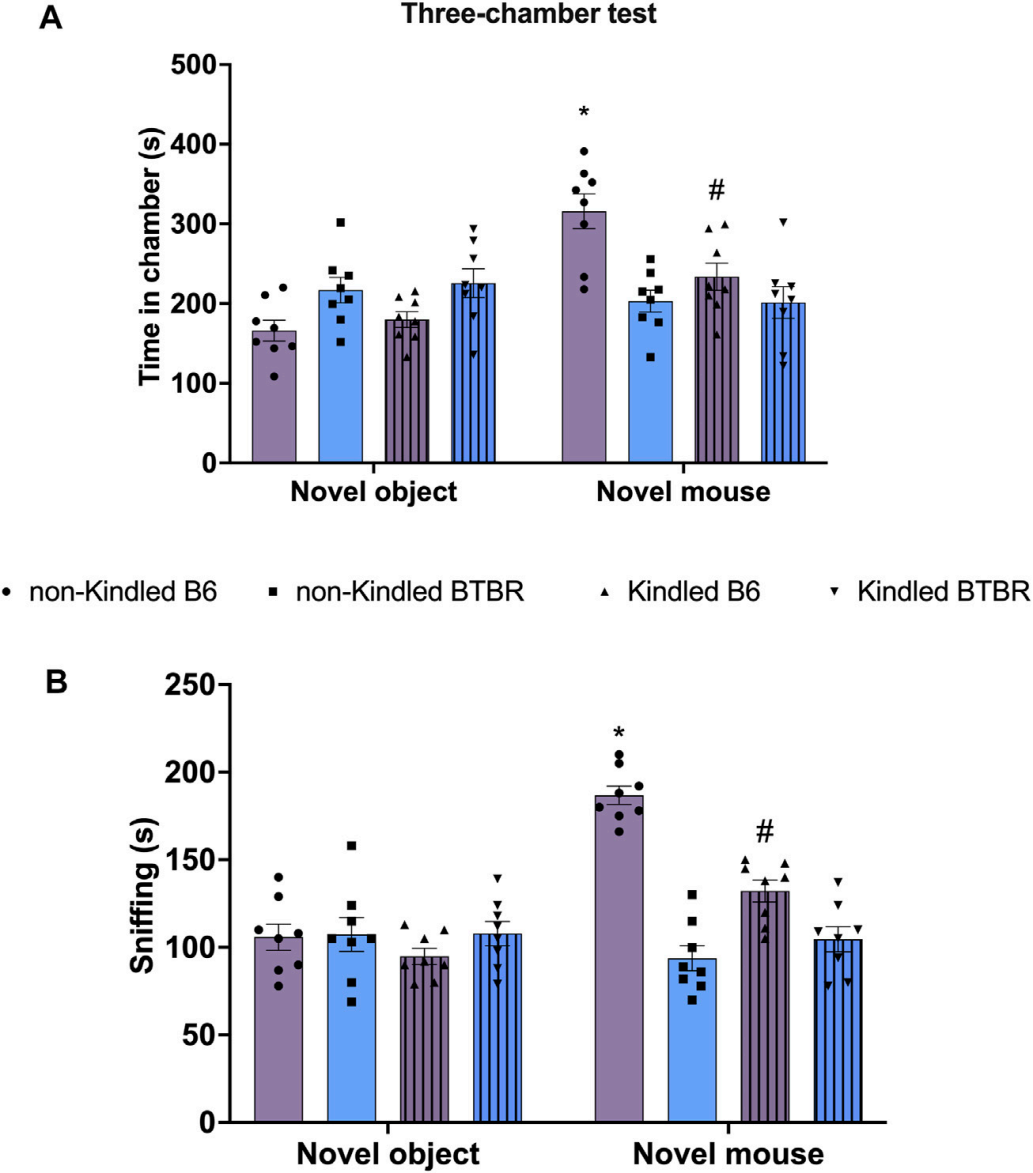
Three chamber social interaction test (TCT) performed at the end of the PTZ-kindling procedure. **(A)** Bars indicate the time (s) spent in the chamber containing the novel object and the time (s) spent in the novel mouse chamber. **(B)** Bars indicate the time (s) spent sniffing the novel mouse. Data are expressed as mean ± S.E.M. ^*^Indicates *p* < 0.0001 vs. novel object, ^#^Indicates *p* < 0.05 vs. non-kindled B6 mice. B6 = C57BL/6J; PTZ = Pentylentetrazole.

Similarly, in TCT, BTBR mice did not exhibit any significant preference for social interaction compared to their respective B6 control group, indicating lower sociability (Figure 3). In detail, the time spent in the novel mouse chamber and in the novel object chamber did not significantly (*p* > 0.05) differ in BTBR mice (Figure 3A). Similarly, the *post hoc* analysis revealed that BTBR mice did not spend more time sniffing the novel mouse than sniffing the novel object (Figure 3B). The sociability was not further impaired after PTZ-kindling in BTBR mice, with these parameters being maintained at a similar level to that shown by non-kindled BTBR mice. At odds, PTZ-kindling significantly (*p* < 0.05) modified sociability in B6 mice. In detail, kindled B6 mice did not exhibit any significant preference for the chamber containing the novel mouse compared to the chamber with the novel object (Figure 3). Furthermore, Tukey’s *post hoc* test revealed that non-kindling B6 mice spent more time in the chamber containing the novel mice compared to kindling B6 mice (Figure 3).

### 3.4 Effects of kindling on anxiety and memory

**FIGURE 4:**
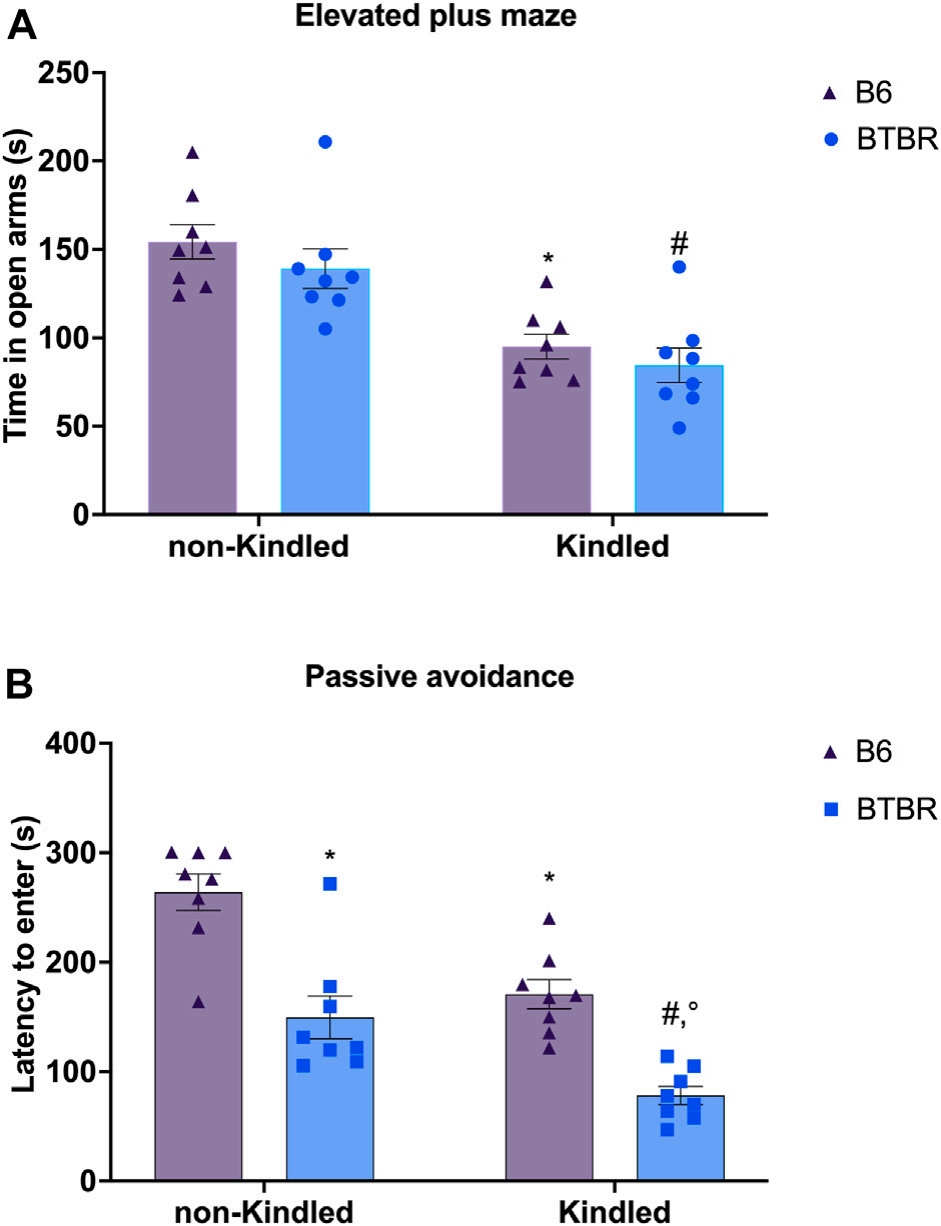
Elevated plus maze (EPM) and passive avoidance (PA) tests performed at the end of the PTZ-kindling procedure. **(A)** Bars indicate time spent in the open arm (s) in the EPM test. **(B)** Bars indicate the latency (s) to enter into the dark chamber in the PA. ^*^ Indicates *p* < 0.05 vs. non-kindled B6 mice, ^#^Indicates *p* < 0.05 vs. non-kindled BTBR mice, ^°^Indicates *p* < 0.05 vs. kindled B6 mice. Data are expressed as means ± S.E.M. B6 = C57BL/6J.

In the EPM, there are no significant differences in the parameters evaluated between BTBR mice and B6 control mice. At odds, PTZ-kindling in BTBR and B6 mice induced a significant reduction in the time spent in the open arms (*p* = 0.026 and *p* = 0.039, respectively), causing an anxious-like behavior in both strains of mice. Furthermore, PTZ-kindling was significantly able to increase the latency to first entry in open arms in BTBR and B6 mice (*p* = 0.0001 and *p* = 0.005, respectively) (Supplementary Figure S1). No significant difference was observed in these parameters between BTBR kindled and B6 kindled mice groups (Figure 4A). In the PA test, BTBR mice have a decreased latency time compared to control B6 mice (*p* = 0.0005), indicating a deficit in learning and memory performance (Leo et al., 2021). This cognitive impairment was significantly (*p* = 0.0073) worsened in the kindled BTBR mice group (Figure 4B). PTZ kindling was also able to significantly impair learning and memory functions in B6 control mice (*p* = 0.0058); however, B6 kindled mice had an increased latency time than kindled BTBR mice (*p* = 0.0006), showing better cognitive performance in this test.

## 4 Discussion

Systematic reviews and meta-analyses have clearly demonstrated that epilepsy and ASD have an overlapping prevalence, suggesting that they possibly share at least some common mechanisms (Pan et al., 2021; Liu et al., 2022; Specchio et al., 2022; Zahra et al., 2022). Regarding this, it has been proposed that the disruption of the balance between E/I neurotransmissions during embryonic and early postnatal brain development could represent one of the pathological events underpinning the co-occurrence of epilepsy and ASD (Richard et al., 2017; Chakraborty et al., 2022; Specchio et al., 2022). This hypothesis is mainly focused on deficits in the GABAergic inhibitory signaling and the consequent hyperexcitability observed in different brain areas such as the cerebral cortex and hippocampus (Bozzi et al., 2018; Chakraborty et al., 2022; Zhao et al., 2022). An abnormal electrical activity due to an imbalance between E/I synaptic transmission is also a leading cause of epilepsy (Devinsky et al., 2018; Cherubini et al., 2022).

Animal models represent an invaluable tool that can be used both to uncover the mechanisms sustaining the co-occurrence of epilepsy and ASD and test potential therapeutic approaches (Sierra-Arregui et al., 2020). In our study, we used the BTBR mouse model of idiopathic autism, which incorporates multiple behavioral phenotypes relevant to all three diagnostic symptoms of ASD in humans. BTBR exhibited selectively decreased social interaction, repetitive behavior, and impaired communication (McFarlane et al., 2008; Meyza and Blanchard, 2017). BTBR mice also display neuropsychiatric-like deficits, such as anxiety and defective cognitive performance, as also reported in ASD patients (Chao et al., 2018).

Herein, we aimed to study the bidirectional link between epilepsy and ASD in the BTBR mouse (Meyza and Blanchard, 2017), in which an imbalance of E/I ratio due to a constitutive impairment of GABAergic neurotransmission contributes to the development and maintenance of their autistic-like behavior (Han et al., 2014; Cellot et al., 2016; Smith et al., 2016; Gandhi and Lee, 2021; Cherubini et al., 2022).

Not surprisingly, in the first experimental design, we demonstrated that BTBR mice displayed an increased seizure susceptibility to chemoconvulsants antagonizing, with different mechanisms of action (Sperk et al., 2004; Mizielinska et al., 2006), GABA_A_ receptors. This phenomenon is consistent with the dysfunction of inhibitory transmission observed in different brain regions in BTBR mice (Han et al., 2014; Cherubini et al., 2022). Moreover, an increased seizure susceptibility to PTZ was previously reported, in adult BTBR mice, after an early postnatal immune challenge with lipopolysaccharide, confirming the presence of a comorbid epileptic phenotype in this strain of mice (Lewis et al., 2018). At odds, Ruskin et al., did not find difference in the seizure score, after PTZ administration (50 mg/kg, i.p.), between BTBR and B6 mice (Ruskin et al., 2013). This contradiction could be linked to the different methods used to elicit and assess seizure susceptibility. Despite the fact that insufficient GABAergic inhibition seems to play a prominent role in facilitating seizures in experimental models of ASD, clinical and pre-clinical studies pointed out that also dysfunctions in glutamatergic transmission can account for the co-occurrence of epilepsy and ASD (Bozzi et al., 2018; Sierra-Arregui et al., 2020; Nisar et al., 2022; Zhao et al., 2022). Regarding BTBR mice, evidence suggested that dysfunctional ionotropic NMDA and AMPA receptors and metabotropic glutamate receptors 5 activity at excitatory synapses could contribute to the pathophysiology of ASD. In fact, treatment with glutamatergic drugs, including AMPAKINE, memantine, and an antagonist of the mGLU5 receptor, has demonstrated favorable outcomes, normalizing ASD-like phenotype in BTBR mice (Silverman et al., 2012; Silverman et al., 2013; Nuzzo et al., 2020; Eissa et al., 2021). However, the possibility that these glutamatergic dysfunctions can influence the E/I balance, facilitating seizure onset, in BTBR mice was poorly investigated. In our study, the CD50 values for different chemoconvulsants acting on the ionotropic glutamate receptors were similar between BTBR and B6 mice, suggesting that the glutamatergic system would seem to maintain its original constitution in BTBR mice at 8 weeks of age. Consistent with this result, immunohistochemical studies demonstrated that the number of glutamatergic synapses and level of excitatory synaptic proteins did not significantly differ between BTBR and B6 mice (Stephenson et al., 2011; Wei et al., 2016), whereas a reduced glutamate release was only found in aged BTBR mice (Wei et al., 2015). By virtue of this, we can hypothesize that the glutamatergic system is not directly responsible for the reduced seizure threshold in BTBR mice. Therefore, it is likely that augmented excitation in BTBR mice is a consequence of the decreased inhibition, mainly due to GABAergic transmission dysfunctions; however, a direct involvement of glutamatergic transmission cannot be excluded (Han et al., 2014). In this regard, further experiments are necessary to clarify the neurobiological role of glutamatergic neurotransmission in this mouse strain.

In agreement with our results, the disruption of E/I homeostasis, and in particular a deficiency in the GABAergic signaling, could represent a reasonable explanation for the increased seizure susceptibility observed in this idiopathic model of ASD (Sierra-Arregui et al., 2020; Zhao et al., 2022). This evidence is in line with several studies carried out in several experimental models of ASD-epilepsy (Cntnap2, En2, Fmr1, Nrp2), in which defects of GABA_A_ receptor-mediated neurotransmission have been related to the pathogenesis of ASD-epilepsy comorbidity (Bozzi et al., 2018). Furthermore, human genetic studies have documented that several mutations of genes regulating the GABAergic system can account for the co-occurrence of ASD-epilepsy comorbidity (Richard et al., 2017; Specchio et al., 2022). To date, little is known about the influence of epilepsy on the core symptoms of ASD, both in patients and in experimental models. Furthermore, epilepsy and ASD can be accompanied by similar neuropsychiatric comorbidities, which negatively affect the phenotypes of these diseases (Specchio et al., 2022). Accordingly, in the second experimental protocol, we aimed to study how epilepsy impacts autistic-like behavior in BTBR mice, performing the PTZ-kindling model. This latter is a gold standard model of chronic epilepsy, epileptogenesis, and epilepsy-associated neuropsychiatric comorbidities (Singh et al., 2021). In our study, repeated injections of sub-convulsive doses of PTZ led to the development of generalized tonic-clonic seizure both in BTBR and B6 mice, without any difference in the seizure score. Surprisingly, BTBR mice displayed an increased latency for the appearance of generalized seizures (stage 5) in comparison to B6 mice, showing reduced sensitivity to PTZ-kindling. Although this partial resistance to PTZ-kindling was previously observed by Ryu et al. (2021), to date, no study has investigated the mechanisms supporting this phenomenon in BTBR mice. It could be suggested that the reduced sensitivity of BTBR mice to PTZ-kindling may be a result of their anatomical and functional alterations in various brain areas, including the striatum, cortex, thalamus, hippocampus, hypothalamus, and corpus callosum (Gandhi and Lee, 2021), involved in the generation and propagation over time of the epileptic activity during the kindling process (Samokhina and Samokhin, 2018). Therefore, these anatomical and functional modifications could delay the etiopathological events underlying the onset and spreading of the kindling process in BTBR mice. This phenomenon could also be related to the age of mice, at the time of PTZ injections, and/or other experimental variables such as the dosing schedule and the route of PTZ administration that can affect the dose and number of injections needed to kindle an animal (Singh et al., 2021). Regarding this, further studies are required in order to justify the difference in response to acute and chronic treatment with PTZ in BTBR mice. PTZ-kindling model is also widely used to study epilepsy-associated comorbidities, such as anxiety-like behavior, sociability, and cognitive impairment (Russo et al., 2013; Zhu et al., 2017; Nesci et al., 2020), which are also strictly linked to ASD phenotype (Napolitano et al., 2022). Accordingly, PTZ-kindled mice have abnormal behaviors comparable to patients with epilepsy and related neuropsychiatric disorders (Singh et al., 2021). As widely reported in the literature, BTBR mice had impaired sociability and an increased repetitive and perseverative behavior, as highlighted by a reduced time spent in the mouse chamber and a higher number of marble-buried compared to control B6 mice respectively, showing an autistic-like behavior (Silverman et al., 2010; Meyza and Blanchard, 2017; Leo et al., 2021). This latter did not worsen in kindled BTBR mice, suggesting a behavior ceiling effect due to a complete maturation of the circuitry underpinning this phenotype, as also previously reported in BTBR mice after an early life immune activation with LPS (Lewis et al., 2018). Interestingly, we documented that kindled B6 mice had impaired sociability in comparison to non-epileptic B6 mice, indicating that seizures can precipitate social approach impairment in the PTZ-kindling model (Takechi et al., 2016; Takechi, 2019).This evidence is in agreement with previous studies indicating that different epilepsy syndromes are often accompanied by behavioral manifestations linked to ASD phenotype, although the type of relation between these neurological disorders is still unclear (Srivastava and Sahin, 2017; Chawner and Owen, 2022; Specchio et al., 2022). Surprisingly, we did not find any difference in repetitive behavior between kindled and non-kindled B6 mice. Several explanations might justify this result, such as a limited sensitivity of the behavioral test to detect slight differences in the performances of B6 mice. In this respect, further experiments also, with other behavioral tests assessing repetitive behavior, are warranted. BTBR model is also characterized by a cognitive decline that can be evaluated in several tests (MacPherson et al., 2008; Leo et al., 2021), and here, we observed, for the first time, that PTZ-kindling was able to worsen BTBR performance in the passive avoidance test, facilitating cognitive decline. Similarly, PTZ kindling has also demonstrated detrimental effects on cognitive performance in B6 mice. In this respect, evidence indicates that epilepsy, neurodevelopmental and cognitive disorders possibly share common underlying mechanisms; however, their precise association still needs to be fully elucidated (Tuchman, 2015; Mikula et al., 2021; Napolitano et al., 2022; Zahra et al., 2022). Very recently, it has been demonstrated that tiagabine suppressed PTZ induced seizures and improved cognitive performance in BALB/c mice, by regulating neurotrophic factors and pro-inflammatory cytokines expression (Javaid et al., 2023).

Regarding anxiety, previous studies detected a large variability in the performance of BTBR mice in the EPM, ranging from the absence of anxiety-like behaviors to anxiety levels similar to B6 mice to heightened behavior in response to stressful events (Benno et al., 2009; Langley et al., 2015; Leo et al., 2021). In this study, PTZ administration altered the behavior of B6 and BTBR mice in the EPM, such that time spent in the open arms was significantly diminished in comparison to non-kindled B6 and BTBR mice, suggesting that both strains of mice may exhibit anxiety-like behavior in response to a stressful event such as PTZ-kindling. This is also in agreement with as previously demonstrated in different strains of mice when this experimental model is performed (Russo et al., 2013; Nesci et al., 2020; Leo et al., 2021).

Overall, the imbalance between E/I in synaptic transmission and neural networks can significantly contribute to the epileptogenic process underlying the reduced seizure threshold observed in BTBR mice. Contrary to our hypothesis, BTBR mice have a partial resistance to develop chronic seizures that could be explained with the PTZ-kindling methodology. Accordingly, the BTBR mouse would seem a valuable model to study the neurobiology of epilepsy-associated ASD; however, to this aim, future studies should shed light on the complex background underpinning the neurological-like disorders observed in the BTBR model.

## 5 Conclusion

Experimental models can be extremely useful in discovering both the causal mechanisms of the co-occurrence of epilepsy and ASD and the potential pharmacological therapies to counteract these disorders. The BTBR mouse has a complex genetic, molecular, and physiological background that deserves further investigation to use this model as a potential tool to study the neurobiology of epilepsy-associated ASD. To this aim, the BTBR mouse model needs to be validated according to the three major criteria: construct, face, and predictive validity (Nestler and Hyman, 2010). BTBR mice have an increased susceptibility to acute seizures induced by GABA antagonists, whereas they achieved the kindled state (3 consecutive stage 5) with a longer latency compared to B6 mice. Interestingly, seizures did not worsen the autistic phenotype in BTBR mice, suggesting a behaviour ceiling effect due to a complete maturation of the circuitry underpinning this phenotype. Since ASDs are neurodevelopmental conditions, we cannot exclude that starting kindling early in BTBR mice life could lead to an additional impact on their autistic phenotype. As also reported by Lucas et al., it is possible that in adolescent mice compensatory mechanisms become active, in the setting of seizure, counteracting the additional deficits. Accordingly, to elucidate the effects of aging, future studies are warranted. This hypothesis would seem to be supported by the fact that seizures, in mice without an abnormal neural substrate, lead to several behavioral changes. At odds, the network underlying absence seizures and cognitive impairment may still undergo further modifications related to aggravation of symptomatology, supporting that different neuronal networks could be involved in these disorders. However, based on the multifactorial etiology of ASD and epilepsy, the causal mechanisms behind this relationship could be many and various, warranting further studies.

Interestingly, BTBR mice have a similar seizure severity score but a longer latency in the development of chemical kindling induced by the administration of PTZ compared to B6 control mice. The present data suggest that the neuroanatomical, biochemical, and electrophysiological deficiency in BTBR mice influences the pathophysiology and pharmacology of acute and chronic epileptic seizures in an opposite manner. However, we cannot exclude that age and/or sex-based differences influence ASD comorbid epilepsy in BTBR mice. This latter point should be addressed to increase our understanding of the underlying mechanisms for the identification of therapeutic targets and improved treatment in both disorders. Future studies should also investigate the susceptibility of BTBR mice in developing chronic seizures using different models other than PTZ-kindling. Likewise, appropriate and validated transgenic models for neurodevelopmental genes involved in epilepsy, such as MeCP2, SYNGAP1, FMR1, SHANK1-3, and TSC1, could increase our knowledge both on the pathogenic basis of epilepsy-associated ASD and the discovery of effective drugs for both disorders.

## Data Availability

The original contributions presented in the study are included in the article/Supplementary Material; further inquiries can be directed to the corresponding author.
