# Supplementary figures and images for: Seizure susceptibility to various convulsant stimuli in the BTBR mouse model of autism spectrum disorders
Source: Front Pharmacol. 2023 Apr 20;14:1155729. doi: 10.3389/fphar.2023.1155729 (PMC10157402; doi:10.3389/fphar.2023.1155729)

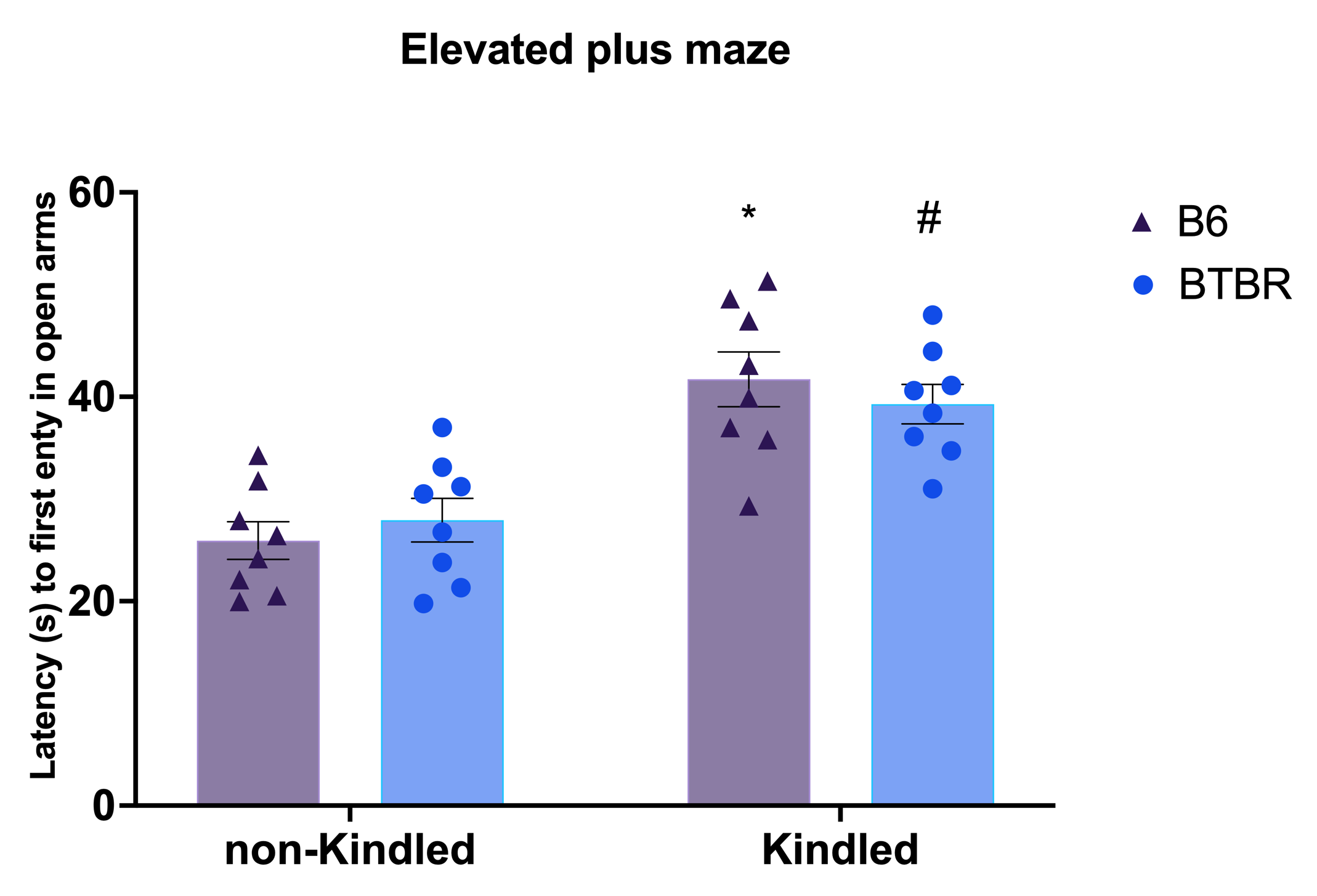

Supplement: Supplementary file 1 [file Image1.TIFF]
